# Delirious Mania Associated with Autoimmune Gastrothyroidal Syndrome of a Mid-Life Female: The Role of Hashimoto Encephalopathy and a 3-Year Follow-Up including Serum Autoantibody Levels

**DOI:** 10.1155/2016/4168050

**Published:** 2016-09-01

**Authors:** Udo Bonnet, Claudia Selle, Ralf Kuhlmann

**Affiliations:** ^1^Department of Psychiatry, Psychotherapy, and Psychosomatic Medicine, Evangelisches Krankenhaus Castrop-Rauxel, Academic Teaching Hospital of the University of Duisburg-Essen, Essen, Germany; ^2^Department of Neurology, Evangelisches Krankenhaus Castrop-Rauxel, Academic Teaching Hospital of the University of Duisburg-Essen, Essen, Germany

## Abstract

We report the case study of a 57-year-old Caucasian female with steroid-responsive encephalopathy associated with autoimmune thyroiditis (SREAT), commonly termed Hashimoto encephalopathy (HE). This presentation includes one of the longest lasting follow-up studies of HE considering the neuropsychiatric symptoms (here delirium, mania, and EEG-slowing) and their relation to serum autoantibody levels. Antithyroid-peroxidase autoantibodies, the hallmark of autoimmune thyroiditis, were found in the serum and also in the cerebrospinal fluid. Diagnostic analyses found no evidence of limbic encephalopathies characterized by serum antibodies against intracellular, synaptic, or further cell surface antigenic targets, neoplasm, and connective tissue or vasculitis diseases. A potential contribution of bipolar disorder and metabolic encephalopathies due to severe hypothyroidism, glucocorticoid treatment, accelerated thyroid hormone replacement therapy, or vitamin B deficiency is critically discussed. Another special feature of this case report is the linkage of HE to an autoimmune polyendocrine syndrome (type 3B) affecting the gastroduodenum in addition to the thyroid gland.

## 1. Introduction

Hashimoto encephalopathy (HE) is a rare pathological condition with an estimated prevalence of 2/100,000 and various neuropsychiatric symptoms (NPS) combined with positive serum antithyroid-peroxidase autoantibodies (TPO-Abs, usually >200 U/mL) [1, 2]. These antibodies are a hallmark of Hashimoto thyroiditis that occurs in 1–5% of the general population [1, 2]. Most patients with HE are euthyroid or have subclinical hypothyroidism, and 20% of them have overt hypothyroidism while hyperthyroidism is rare [1, 2]. HE's clinical onset is usually subacute and its clinical presentation is multifaceted including neuropsychiatric symptoms (NPS), such as cognitive decline, behavioral symptoms, depression, psychosis, cerebral ischemia, seizure, myoclonus, tremors, and fluctuating levels of consciousness [1, 2]. Abnormalities in neuroimaging, electroencephalogram (EEG), and cerebrospinal fluid (CSF) are not required to diagnose HE although being present in more than 50% of the cases (usually elevated CSF protein, generalized slowing of EEG-waves, and T2-hyperintense foci on brain MR imaging) [1, 2]. HE is characterized by a remission of NPS, followed by normalization of neuroimaging and EEG after corticosteroid (glucocorticoid) therapy (steroid-responsiveness) or, if resistant, escalating immunosuppression [1, 2]. However, cases with partial remission or progression even up to death were described [1]. Data regarding the course of HE are scarce and rely on clinical follow-ups which usually lasted from 6 to 24 months. A review of the 82 cases reported in literature revealed that, in this time span, most HE patients (>50%) had not relapsed while 30% had relapsed after glucocorticoid discontinuation, 5% of whom had died [1]. The etiology of HE is obscure. Speculations about the pathogenesis include autoimmune cerebral vasculitis, neurotoxic effects of thyroid-stimulating hormone, or autoantibodies [1–3]. Regarding HE's higher prevalence in women (80% of the cases), fluctuating course, and good response to steroids, an autoimmune mechanism is most likely [1]. It is assumed that thyroid autoantibodies did not play a causative role in producing HE's brain dysfunction and are simply “bystanders” of the disease because their serum or CSF titers were found to be not associated with the severity of NPS, and incidental serum TPO-Abs were common [1, 2]. Moreover, these autoantibodies were not consistently found in the CSF of HE patients [4]. However, TPO-Abs were demonstrated to bind to astrocytes and it was suggested that they disturb CNS-function [3]. Moreover, a couple of clinical improvements were shown to be accompanied by a decrease of serum TPO-Ab titers [1, 2]. Higher serum TPO-Ab titers were correlated with better outcomes [5]. However, more long-term clinical observations considering the relationship between clinical outcome and antithyroid autoantibody titers are warranted.

The special features of the following case report are (i) presumably the first association of an intricate delirium and mania (delirious mania) with HE, (ii) relation to an autoimmune polyendocrine syndrome, (iii) a 3-year clinical follow-up including the measurement of serum antithyroid autoantibodies, and (iv) a critical discussion of a potential contribution of differential diagnoses, such as bipolar disorder, other limbic or metabolic encephalopathies, the latter ones due to hypothyroidism, glucocorticoids, accelerated thyroid hormone replacement therapy, and vitamin B deficiency.

## 2. Case Report

### 2.1. History and Diagnostic Analyses

In the past year, a 57-year-old Caucasian female (61 kg, 166 cm) felt gradually empty, exhausted, tired, and lethargic. In addition, she perceived constipation and cold intolerance, which was accompanied by a decline of appetite, concentration, and productivity. She attributed her complaints to the growing level of distress at her work as a teacher and preferred to watch and wait without consulting a physician. Apart from that, her medical, addiction, and familial history were unremarkable. In the last four weeks, she developed a state of apathy and mutism combined with fluctuating confusion, amnesia, disorganization, and incontinence, which led to hospital treatment that was initiated by her husband. Upon emergency admission, a hypoactive delirium and severe hypothyroidism were diagnosed (Table 1). Physical and neurologic examination showed signs of hypothyroidism (shortness of breath, bradycardia, hypothermia, swollen limbs, dry skin, delayed relaxation of tendon reflexes, and myalgia) [8]. Transabdominal echocardiography found pericardial effusion [8]. Routine lab checks revealed further signs of hypothyroidism, such AST- and CK-elevations and hypercholesterinemia (Table 1) [8]. Alcohol intoxication and hyperammonemia were excluded. Moreover, macrocytic anemia was found together with slight hyperhomocysteinemia (12.4 *μ*mol/L, reference level <9 *μ*mol/L), but without laboratory vitamin B deficiency (normal serum levels of vitamins B1, B6, B9, and B12, methylmalonic acid, and holotranscobalamin). Vitamin D was found to be slightly reduced and substituted then. The EEG was slightly slowed (Table 1). The brain MR imaging showed a few unspecific T2-hyperintense areas in the white matter (WMH) of both temporal lobes, which could be compatible with severe hypothyroidism, vasculitis, or autoimmune encephalopathy. In the serum, elevated TPO-Abs (Table 1) were found which pointed to Hashimoto thyroiditis [1, 2, 8] being responsible for the hypothyroidism. This was underlined by an atrophic thyroid gland in ultrasound. Regarding the remaining serum antithyroid autoantibodies, TSH-receptor autoantibodies (TR-Abs, Table 1) were also elevated and thyroglobulin autoantibodies were normal. Later on, elevated autoantibodies against parietal cells (PC-Abs, Table 1) and intrinsic factor (11.43 U/mL, normal <1.5 U/mL) were found, which most likely corresponded to a helicobacter pylorus-negative lymphocytic gastroduodenitis in histopathology (up to 80 intraepithelial lymphocytes per 100 epithelial nuclei, T-lymphocytes) and macrocytic anemia. The lymphocyte typing of the blood found increased T-helper-lymphocytes (1350/*μ*L, normal 645–1289/*μ*L), cytotoxic T-lymphocytes within its normal range, and no evidence of a cellular immune deficiency as being measured in the last asymptomatic month (Table 1). Helicobacter pylori antibodies were negative. There were no clinical or immunologic signs of Addison's disease, diabetes mellitus, vitiligo, alopecia, or celiac disease. Thus, outside the CNS, the autoimmune disease appeared to be limited to a gastroduodenal-thyroidal syndrome, classified as autoimmune polyendocrine syndrome type 3B [9]. Other forms of limbic encephalopathies that could have been associated with antibodies against intracellular, synaptic, or other cell surface antigenic targets [10], neoplasm, connective tissue, or vasculitis diseases [1, 2] were excluded.

In the CSF, TPO-Abs (33.8 IU/mL, normal: 0–8 IU/mL) [4] were detected, while TR-Abs and thyroglobulin antibodies were not investigated. Moreover, CSF protein (43.7 mg/dL, normal 15–35 mg/dL), S-100 protein (3200 pg/mL, normal <2700 pg/mL), and neuronal specific *α*-enolase (NSE, 16.9 *μ*g/L, normal <13 *μ*g/L) were elevated. Pleocytosis or oligoclonal bands were missing. Also, neurodegeneration markers (beta-amyloid, tau-, and phospho-tau protein), angiotensin converting enzyme (ACE), lysozyme, titers of immunoglobulins (IgM, IgA, and IgG), IgM-, IgA-, and IgG-indices, antibody index for either herpes simplex virus, varicella zoster virus, cytomegalovirus, measles virus, rubella virus, or* Borrelia burgdorferi*, and 14-3-3 protein were unremarkable in the CSF.

Ophthalmological, angiological, and nephrological examinations were unremarkable, too. Subsequent electrocardiograms, abdominal ultrasound, chest X-ray, and the remaining serum routine lab including ferritin and transferrin were normal as well as immunoglobulins including subclasses, carbohydrate deficient transferrin, parathyroid hormone, lysozyme, ACE, carbohydrate deficient transferrin, gastrin, and serum autoantibodies against thyroglobulin and the amino terminal of *α*-enolase (NAE). Virus hepatitis, HIV, herpes, syphilis, Lyme disease, and M. Whipple were excluded. Drug urine screen was negative.

### 2.2. Course of the Treatment

Regarding the increasing somnolence and falling heart rate (down to 40 bpm) and body temperature (down to 33.8°C) the critical care management included an accelerated thyroid hormone replacement therapy (THRT) with L-thyroxine (IV, 250 *μ*g/day in the first 5 days) and IV glucocorticoids (125 mg/day IV methylprednisolone) to prevent the progression of the hypoactive delirium to myxedema coma [11]. In the next few days, the critical condition improved. Hypothermia and bradycardia resolved within 36 hours and thyroid-stimulating hormone (TSH) started to fall. Simultaneously, the delirium turned into a mixed state (Table 1), requiring low doses of haloperidol (up to 10 mg/day) and clonazepam (up to 1 mg/day) to cope with arising visual or auditory hallucinations and agitation.

When the results of elevated serum TPO-Abs came in, the IV glucocorticoid treatment was enhanced to 250 mg/day IV methylprednisolone assuming that the delirium was based on HE [1, 2]. Thereafter, the mixed delirium (fluctuating vigilance, disorientation, disorganization, starring, memory impairment, irritability, hallucinations, and delusion of reference) turned to a hyperactive state with striking manic features (euphoria, grandiosity, racing thoughts, sleeplessness, and risky and reckless behavior). Meanwhile, the patient's physical condition had been stabilized and she was voluntarily transferred from the critical care unit to the psychiatric ward. Assuming that the glucocorticoids had evoked the mania, the prednisolone dose was reduced to 25 mg/day which was followed by a worsening of both delirium and mania (Table 1). A subsequent pulse therapy of 1000 mg/day IV methylprednisolone over 3 days was associated with a prompt resolution of the delirium and improvement of the mania (Table 1). As the mania did not completely resolve along with oral prednisolone (100 mg/day), the treatment was augmented with quetiapine (up to 300 mg/day). The patient became euthymic within the next 3 weeks and could be transferred to an outpatient treatment (Table 1). Quetiapine and prednisolone were tapered within the subsequent 4 and 24 weeks, respectively, without changing the normal mental and physical health condition under THRT alone, which is still continuing up until now, 3 years later (Table 1). No further medications were required. Vitamin D supplementation was stopped after 6 months and the 25-OH-vitamin D-levels were normal in the controls carried out every six months.

Serum antithyroid autoantibody titers respond to the glucocorticoid treatment without a timely correlation with the patient's mental status (Table 1). TR-Abs sustainably normalized while TPO-Abs gradually increased after glucocorticoid taper, however, without a worsening of her normal mental status (Table 1). Simultaneously, the THRT could be reduced (Table 1). Macrocytic anemia resolved although the PC-Ab titers increased progressively (Table 1) and antibodies against the intrinsic factor were found. Vitamin B levels, holotranscobalamin, and homocysteine were controlled every six months and remained normal. Brain MR imaging and EEG normalized under the combined glucocorticoid and thyroid hormone treatment, which corresponded to the resolution of delirious mania (Table 1). Furthermore, brain MR imaging and EEG were normal in the controls carried out 12 and 30 months after termination of the glucocorticoid treatment.

## 3. Discussion

This is the first case report which describes the coexistence of a delirium and mania in association with HE. HE was clinically specified according to the following commonly used criteria: (i) encephalopathy signs (cognitive decline and further NPS), (ii) presence of serum TPO-Abs (>200 U/mL), (iii) exclusion of infectious, metabolic, paraneoplastic, or toxic etiologies (by serum, CSF, and brain imaging), and (iv) marked improvement of symptoms after administration of steroids or immunosuppression [1, 2]. HE was further specified by the exclusion of other forms of limbic encephalopathies [10]. TPO-Abs were also present in CSF [4] while serum NAE autoantibodies which are reported to be often associated with HE [12] were not found. Characteristically, HE appears in middle-aged females [1, 2], just as reported here.

Of note, some mental symptoms of the patient can be alternatively explained by severe hypothyroidism (preceding depression, hypoactive delirium, mania, elevated CSF protein, EEG-slowing, and WMH) [13–15] or rapid adjustment of THRT (hyperactive delirium and mania) [16]. A direct influence of glucocorticoid exposure to the development of delirious mania is unlikely because this condition improved during steroid pulse therapy. The amount of serum TPO-Abs was not related to NPS, on first sight supporting the assumption that these autoantibodies were not causally involved in the generation of NPS of HE [1, 2]. Considering the atrophic gland in thyroid ultrasound, it is likely that serum TPO-Abs had been elevated already a couple of years prior to the manifestation of overt hypothyroidism and delirious mania. However, there are TPO-Abs even in our patient's CSF which have been related to cytotoxicity by some authors [3, 4]. In the patient's CSF, these autoantibodies could have developed more recently due to sensitivity changes of her immune defense during the period of marked occupational distress prior to her malaise. There is evidence that the synthesis of intrathecal TPO-Abs takes place in the CNS itself [4] which could not be confirmed in the presented case report regarding the patient's normal IgG-index and missing oligoclonal bands in CSF.

Her severe hypothyroidism might be involved in the development of HE via disturbing the integrity of blood brain barrier, which could have facilitated the transfer of putatively cytotoxic [3, 4] TPO-Abs from blood stream into brain tissue. Remarkably, signs of glial and neuronal cell injury were found in our patient's CSF (elevated S-100 protein and NSE). Vitamin D-deficiency could have promoted the development of Hashimoto thyroiditis [17]. An ongoing, while smoldering, autoimmune process [9] can be assumed from accessory, actually asymptomatic, autoimmune gastritis and lymphocytic colitis with elevated T-helper cells and rising PC-Abs in her serum. Therefore, the therapy focuses on strengthening her coping with distress and relieving the strain on her.

Interestingly, elevated TR-Abs were found in our patient's serum which had been observed in some HE cases by other authors, too [12]. Taking into account that the supplementation of thyroid hormone could be reduced while TR-Ab activity had normalized (Table 1), these TR-Abs were most likely of the TSH-receptor blocking type [18].

Regarding the alternative explanations for cerebral biomarkers that have been observed and associated with HE in this case report, EEG-slowing and WMH were demonstrated to be positively correlated with serum TSH-levels [14–16]. However, an influence of antithyroid antibodies was not excluded in these studies [14–16]. Furthermore, WMH was described to correspond with hyperhomocysteinemia [19], and a positive relationship between serum TSH and homocysteine was reported [20].

Possibly, the IV administration of L-thyroxine, which was used to prevent a progression from myxedema to coma, could have forced the manic state [16]. A 3-year follow-up documenting a normal psychiatric condition under THRT alone may point to an association of our patient's delirious mania with thyroid dysfunction.

Regarding her psychopathology, there were some striking catatonic symptoms (mutism, starring, and agitation) being frequently reported in patients with delirious mania [21]. Catatonia was observed in both delirium of encephalopathies, for example, based on severe hypothyroidism or HE, and severe mania in bipolar disorder [21]. At this juncture, serum TPO-Abs were found more frequently in patients with bipolar disorder if compared with other patients and, moreover, were related to the genetic vulnerability to develop bipolar disorder [22]. Personal and family history of our patient was unremarkable in this regard.

Over three years, our patient's serum TPO-Ab titers were not associated with NPS. A similar long-term observation was recently published, describing a 61-year-old Caucasian woman diagnosed with HE, however, being continuously under immune suppression [23], which was not required in our patient. The authors are unaware of further long-term follow-ups to this subject.

In conclusion, HE is an ambiguous and causally interwoven clinical condition. Only the fast steroid-responsiveness of NPS (here delirium and subsequent mania) and EEG-slowing remained to be specific to HE in the presented case report. This is hardly different from other case reports about HE [1, 2].

## Figures and Tables

**(a):** 

Treatment day	Clinical features	ICDSC^*∗*^-points	YMRS^*∗∗*^-points	EEG-rhythm	TSH (0,4–4,0 *μ*U/mL)	fT3 (3,5–6.5 pmol/L)	fT4 (11,5–22,7 pmol/L)	TPO-Abs (<34 IU/L)	TR-Abs (<1,75 IE/L)	PC-Abs (<1/10 titers)

*1*	**Hypoactive delirium**	**7**	n/a	**7.5/s**	**91.44**	**<0,3**	**2.40**	n/a	n/a	n/a
*3*	**Mixed delirium**	**7**	n/a	n/a	**66.61**	**1.30**	**7.23**	**334.00**	**24.00**	n/a
*5*	**Mixed delirium**	**5**	n/a	11/s	**50.25**	**1.60**	**9.00**	n/a	n/a	n/a
*9*	**Mixed delirium**	**4**	n/a	10/s	**43.50**	**2.60**	**13.96**	n/a	n/a	n/a
*15*	**Hyperactive delirium, euphoric mania**	**4**	**39**	n/a	**38.80**	3.60	19.90	n/a	n/a	n/a
*22*	**Euphoric mania**	3	**24**	**8/s**	**20.57**	3.60	20.26	**323.00**	**26.96**	n/a
*26*	**Hyperactive delirium, euphoric mania**	**6**	**47**	**8/s**	**17.27**	n/a	n/a	**274.00**	**24.73**	n/a
*29*	**Euphoric mania**	3	**39**	**7,5/s**	2.52	4.10	24.45	n/a	n/a	n/a
*36*	**Euphoric mania**	3	**33**	**8/s**	4.18	4.30	23.96	**249.00**	**21.14**	n/a
*43*	**Hypomania**	0	**12**	10,5/s	2.30	3.70	19.99	**212.00**	n/a	n/a
*50*	**Hypomania**	0	**8**	9,5/s	1.03	n/a	n/a	**208.00**	n/a	n/a
*57*	Normal	0	2	n/a	**9.90**	n/a	n/a	**188.00**	**55.10**	n/a
*64*	Normal	0	1	n/a	**11.69**	4.10	19.46	n/a	n/a	**1/80**
71	Normal	0	1	n/a	**15.15**	4.40	19.92	n/a	n/a	n/a
78	Normal	0	0	9/s	4.72	4.60	22.66	**159.00**	**8.71**	**1/80**
83	Normal	0	0	n/a	3.24	4.70	23.72	**165.00**	**8.40**	n/a
90	Normal	0	0	9,5/s	**5.08**	n/a	n/a	**139.00**	n/a	n/a
119	Normal	0	0	10/s	0.11	n/a	n/a	**135.00**	**4.69**	n/a
140	Normal	0	0	10/s	**0.03**	**6.90**	**30.16**	**152.00**	**5.00**	**1/320**
176	Normal	0	0	9/s	n/a	n/a	n/a	**172.00**	**5.65**	n/a
261	Normal	0	0	11/s	**0.01**	6.40	**32.55**	**229.00**	**6.16**	**>1/1280**
323	Normal	0	0	9,5/s	**0.10**	22.60	4.70	**226.00**	**4.52**	**>1/1280**
367	Normal	0	0	9,5/s	0.51	4.50	**23.22**	**246.00**	**2.69**	**>1/1280**
437	Normal	0	0	9,5/s	**0.05**	6.3	**24.68**	**261.00**	n/a	**>1/1280**
595	Normal	0	0	n/a	**0.39**	4.5	**22.99**	**337.00**	0.37	**>1/1280**
842	Normal	0	0	9,5/s	0.98	n/a	n/a	**291.00**	<03	**>1/1280**
1104	Normal	0	0	10/s	1.83	4.10	16.35	**348.00**	0.47	**>1/1280**
1264	Normal	0	0	10/s	2.96	4.00	16.12	**345.00**	0.39	**>1/1280**

**(b):** 

Treatment day	Clinical features	Prednisolone (mg/day)	L-Thyroxine (*μ*g/day)	RBC (4.1–5.1 million cells/mcL)	Hemoglobin (12–18 g/dL)	MCV (77–91 fL)	Lipase (73–393 U/L)	AST (0–35 U/L)	LDH (0–249 U/L)	CK (0–190 U/L)	Cholesterol (0–260 mg/dL)
*1*	**Hypoactive delirium**	125 IV^*∗∗∗*^	250 IV	**3.27**	**10.50**	**95.10**	230.00	**89.00**	**898.00**	**3358.00**	**766.00**
*3*	**Mixed delirium**	250 IV^*∗∗∗*^	150	**2.85**	**8.90**	**96.00**	218.00	**47.00**	**597.00**	**1899.00**	n/a
*5*	**Mixed delirium**	100	150	**2.79**	**8.80**	**95.80**	**499.00**	**46.00**	**591.00**	n/a	n/a
*9*	**Mixed delirium**	50	150	**2.96**	**8.80**	**97.20**	**582.00**	28.00	**589.00**	**200.00**	n/a
*15*	**Hyperactive delirium, euphoric mania**	50	150	**3.11**	**9.80**	**98.80**	**836.00**	16.00	**497.00**	92.00	n/a
*22*	**Euphoric mania**	25	150	**3.13**	**9.90**	**98.10**	189.00	7.00	**292.00**	31.00	**512.00**
*26*	**Hyperactive delirium, euphoric mania**	1000 IV^*∗∗∗*^	150	n/a	n/a	n/a	n/a	n/a	n/a	n/a	n/a
*29*	**Euphoric mania**	100	150	**3.28**	**10.20**	**96.00**	345.00	6.00	n/a	24.00	**530.00**
*36*	**Euphoric mania**	60	150	**3.32**	**10.40**	**95.90**	283.00	8.00	227.00	24.00	**383.00**
*43*	**Hypomania**	50	150	**3.50**	**10.50**	**95.20**	183.00	7.00	218.00	18.00	**294.00**
*50*	**Hypomania**	30	150	**3.16**	**9.50**	**93.50**	217.00	10.00	202.00	20.00	251.00
*57*	Normal	25	150	**3.58**	**10.70**	**95.30**	204.00	11.00	236.00	21.00	**268.00**
*64*	Normal	15	150	**3.76**	**11.00**	**94.60**	n/a	n/a	n/a	n/a	n/a
71	Normal	10	175	**3.66**	**10.90**	**93.90**	183.00	13.00	210.00	28.00	n/a
78	Normal	8	175	**3.55**	**10.40**	**92.10**	n/a	n/a	213.00	n/a	n/a
83	Normal	8	175	**4.09**	12.00	90.30	212.00	11.00	203.00	34.00	n/a
90	Normal	5	175	**4.03**	**11.90**	89.00	n/a	n/a	n/a	n/a	243.00
119	Normal	5	175	4.12	**11.50**	89.30	n/a	n/a	n/a	52.00	n/a
140	Normal	5	175	4.26	**11.90**	87.40	n/a	14.00	n/a	60.00	n/a
176	Normal	3	150	n/a	n/a	n/a	n/a	n/a	n/a	n/a	n/a
261	Normal	1	100	4.40	12.60	87.30	n/a	21.00	n/a	86.00	**317.00**
323	Normal	0	87.50	4.13	**11.80**	88.10	129.00	16.00	n/a	106.00	**265.00**
367	Normal	0	87.50	4.45	13.00	**92.40**	n/a	14.00	n/a	n/a	n/a
437	Normal	0	87.50	4.31	12.8	**91.6**	n/a	17	n/a	n/a	n/a
595	Normal	0	87.50	4.24	13	**91.5**	144	20	n/a	100	**295**
842	Normal	0	75	4.21	12.4	89.7	n/a	16	n/a	87	**290**
1104	Normal	0	75	4.29	12.8	90.1	159	11	208	68	**296**
1264	Normal	0	75	4.33	12.8	90.8	140	9	205	108	**301**

^*∗*^YMRS (normal ≤ 5, mildly manic 5–19, moderately manic 20–29 points, severely manic ≥ 30 points) [6]. ^*∗∗*^ICDSC: ≥4 points = delirium, 1–3 points = suspicion of subsyndromal delirium, 0 points = no delirium [7]. ^*∗∗∗*^Methylprednisolone. Italic numbers indicate inpatient treatment days. Days 1–14: intensive care unit, days 15–28 and days 29–64 closed and open psychiatric ward, respectively. Afterwards, outpatient treatment. TSH (thyroid-stimulating hormone), free thyroxine (fT4), free triiodothyronine (fT3), TPO-Abs (thyroid-peroxidase autoantibodies), TR-Abs (TSH-receptor autoantibodies), PC-Abs (autoantibodies against parietal cells). Normal ranges of serum lab results in brackets. Pathological results in bold letters/numbers.
